# Characterization of the Phosphoproteome in SLE Patients

**DOI:** 10.1371/journal.pone.0053129

**Published:** 2012-12-28

**Authors:** Xinzhou Zhang, Hualin Ma, Jianrong Huang, Yong Dai

**Affiliations:** 1 Department of Nephrology, Shenzhen People's Hospital, Second Clinical Medical College, Jinan University, Shenzhen, China; 2 Clinical Medical Research Center, Shenzhen People's Hospital, Second Clinical Medical College, Jinan University, Shenzhen, China; University of Florida, United States of America

## Abstract

Protein phosphorylation is a complex regulatory event that is involved in the signaling networks that affect virtually every cellular process. The protein phosphorylation may be a novel source for discovering biomarkers and drug targets. However, a systematic analysis of the phosphoproteome in patients with SLE has not been performed. To clarify the pathogenesis of systemic lupus erythematosus (SLE), we compared phosphoprotein expression in PBMCs from SLE patients and normal subjects using proteomics analyses. Phosphopeptides were enriched using TiO_2_ from PBMCs isolated from 15 SLE patients and 15 healthy subjects and then analyzed by automated LC-MS/MS analysis. Phosphorylation sites were identified and quantitated by MASCOT and MaxQuant. A total of 1035 phosphorylation sites corresponding to 618 NCBI-annotated genes were identified in SLE patients compared with normal subjects. Differentially expressed proteins, peptides and phosphorylation sites were then subjected to bioinformatics analyses. Gene ontology(GO) and pathway analyses showed that nucleic acid metabolism, cellular component organization, transport and multicellular organismal development pathways made up the largest proportions of the differentially expressed genes. Pathway analyses showed that the mitogen-activated protein kinase (MAPK) signaling pathway and actin cytoskeleton regulators made up the largest proportions of the metabolic pathways. Network analysis showed that rous sarcoma oncogene (SRC), v-rel reticuloendotheliosis viral oncogene homolog A (RELA), histone deacetylase (HDA1C) and protein kinase C, delta (PRKCD) play important roles in the stability of the network. These data suggest that aberrant protein phosphorylation may contribute to SLE pathogenesis.

## Introduction

Protein phosphorylation is a widespread post-translational modification (PTM). Reversible protein phosphorylation, in which phosphate groups are enzymatically added by protein kinases and removed from proteins by phosphatases, often serves as a molecular switch in signaling pathways. Disruptions in phosphorylation-mediated cell signaling events are connected with numerous diseases [1], [2], [3], [4], [5]. Furthermore, the abnormal expression of protein kinases is an important cause or component of many pathologies. Therefore, the characterization of the phosphorylation sites of proteins within various signaling pathways can enhance the understanding of specific disease pathologies [6]. Phosphoproteomics is defined as the study of the components of the proteome that undergo phosphorylation. Systemic lupus erythematosus (SLE) is a classical autoimmune disease. The disease incidence is nine times greater in women than in men [7], and its estimated prevalence in China is 37.7/100,000 persons [8]. However, the details of SLE etiology remain poorly understood. In this study, we thoroughly explored the phosphopeptide proteome of human Peripheral blood mononuclear cells (PBMCs) using a highly sensitive Liquid chromatography-mass spectrometry (LC-MS/MS) system, improved software for phosphopeptide identification and subsequent analysis with an elaborate bioinformatics strategy, including gene ontology (GO) analysis, pathway analysis and protein network analysis. The rich data from the proteomic analysis also provides insight into the pathogenesis of SLE.

## Materials and Methods

### Patient Assessments and Classifications

This study protocols and consent forms were approved by the Second Clinical Medical College (Shenzhen People’s Hospital) of Jinan University and adhere to the Helsinki Declaration guidelines on ethical principles for medical research involving human subjects. Written informed consent was obtained from all participants. A group of 15 SLE patients who had never been treated with disease-modifying antirheumatic drugs (DMARDs) or other immunosuppressive drugs was recruited for this study. Patients treated with nonsteroidal anti-inflammatory drugs or other symptomatic treatments were excluded. All patients satisfied the American College of Rheumatology classification criteria for SLE. In addition, we choose 15 healthy subjects as controls.

### PBMCs Isolation and Protein Extraction

Peripheral blood mononuclear cells (PBMCs) were separated by a Ficoll-Paque (Sigma, St. Louis, MO, USA) density gradient centrifugation according to the manufacturer’s instructions. In brief, 2 ml blood (with EDTA as an anticoagulant) was layered on 3 ml Ficoll-Hypaque (Sigma) and centrifuged for 25 min at 1300 rpm at room temperature. Mononuclear cells at the interface were aspirated with a Pasteur pipette, washed twice in PBS with centrifugation for 10 min at 900 rpm at room temperature and resuspended in 500 µl SDT lysate (Invitrogen, Carlsbad, USA). The samples were then stored at −80°C until further use.

### Phosphopeptide Enrichment

Phosphopeptides from digested peptides were enriched using the Phosphopeptide Enrichment TiO_2_ kit (Calbiochem, San Diego, CA) according to the manufacturer's instructions. Briefly, the tryptic digest was dried, dissolved in 200 µL TiO_2_ Phosphobind buffer containing 50 g/L 2,5-dihydroxybenzoic acid (DHB) and then mixed with 50 µL TiO_2_ Phosphobind Resin. After a 40 minute incubation, the supernatant was discarded, and the TiO_2_ resin was washed twice with the washing buffer. Then, elution buffer was added to elute the phosphopeptides in two batches. The eluted supernatant was pooled and dried by evaporation for LC-MS/MS analysis.

### LC-MS/MS Analysis

The dried phosphopeptides were subjected to LC-MS/MS analysis with a Finnigan Surveyor High Performance Liquid Chromatography(HPLC) system coupled with a LTQ-Orbitrap XL mass spectrometer (Thermo Electron, San Jose, CA). Briefly, the peptide mixtures were loaded onto a C18 column (100 µm i.d., 10 cm long, 5 µm, resin from Michrom Bioresources, Auburn, CA) using an autosampler. Peptides were eluted in a 5–35% gradient of buffer solution over 180 min and then detected in the LTQ-Orbitrap XL mass spectrometer as described previously [9], [10].

### Raw MS Data Analysis

Raw Orbitrap full-scan MS and ion trap MSA spectra were processed using the MaxQuant algorithms [11], [12]. Peptides and proteins were identified by Mascot through automated database matching of all tandem mass spectra against an in-house curated concatenated target database. Scoring was performed in MaxQuant as described previously. We required strict trypsin enzyme specificity and allowed up to two missed cleavage sites. Cysteine carbamidomethylation (Cys, +57.021464 Da) was searched as a fixed modification, whereas N-acetylation of proteins (N-terminal, +42.010565 Da), oxidized methionine (Met,+15.994915 Da), and serine, threonine, and tyrosine phosphorylations (Ser/Thr/Tyr, +79.966331 Da) were searched as variable modifications.

### Peptide filtering and Phosphorylation Site Identification

The Mascot result files were imported into the MaxQuant software suite for further processing. In MaxQuant, we defined the estimated false discovery rate (FDR) of all peptide and protein identifications at 1% by automatically filtering based on peptide length, mass error precision estimates, and the Mascot scores of all forward and reversed peptide identifications. The final estimate of true phosphorylated amino acids remaining within all identified phosphopeptide sequences was calculated in MaxQuant based on the localization probabilities of all assumed threonine, serine and tyrosine phosphorylation sites using the PTM score algorithm, as described previously [13].For protein identification, we used IPI database. A protein group was removed if all identified peptides assigned to this protein group were also assigned to another protein group. Tosort out a single protein member from a protein group, we chose the protein from the Swiss-Prot database and with the highest sequence coverage. When using label-free approach to identify differently expressed protein and calculating the coefficient of variance, the number of spectra of each protein was logarithmtransformed.

### Different Gene Screening and Statistical Analyses

For screening of phosphorylation sites between the two groups, we used the following method. 1, caculate the fold change between the two groups. 2. set threshold value is 1, that is the average fold change between SLE patients group and healthy controls group was more than or equal to 2 folds; and the *p* value of single sample t-test was less than or equal to 0.05. T-test was conducted using MATLAB 7.5. 3. labeling the gene name corresponding protein according to the NCBI annotation information.

### Bioinformatics Analysis

The expression values calculated for the differential proteins and peptides were used for distance and average to determine linkage for gene ontology (GO) analysis. In pathway analysis, interactions between genes in the range of genomes were analyzed by downloading the pathway data in Kyoto Encyclopedia of Genes and Genomes(KEGG). Finally, the results of the above data were merged into a comprehensive gene inter-relationship network. The established gene network was able to directly reflect the inter-relationships between genes at a whole-cell level, as well as the stability of the gene regulatory network.

## Results

### The Clinical Characteristics of the Study Population

A total of 30 subjects were in the study group, which included 15 SLE patients and 15 healthy controls. In Table 1, the clinical characteristics of the study population are summarized.

**Table 1:** Demographic and disease manifestation in SLE patients and healthy controls.

characteristic	SLE patients	healthy controls
NO.Female/male	15/0	15/0
Age mean (range) years	33.8(19–54)	33.1(21–55)
SLEDI score	12.2	N/A
Disease manifestation		
Skin rash	3	0
Mucosal ulcers	2	0
Proteinuria	5	0
Hematuria	1	0
Arthritis	4	0
Vasculitis	1	0
Low complement	7	0
Increased dsDNA	8	0
Pericarditis	1	0
Leukopenia	2	0
N/A: not applicable		

### Pretreatment of the Raw Data and Screening of Different Genes

A phosphorylated peptide reagent kit was used to enrich the sample for phosphorylated proteins, thus combining protein separation enrichment technology and mass spectrometry technology. The detailed information on the identified phosphoproteins/phosphopeptides according to the mass spectrometric results (Table S1). A total of 1035 phosphorylation sites, corresponding to 618 NCBI-annotated genes, were identified as differentially modified in SLE patients compared with normal subjects.

### GO Annotation and Analysis of the Differences in Phosphoproteins

**Figure 1:**
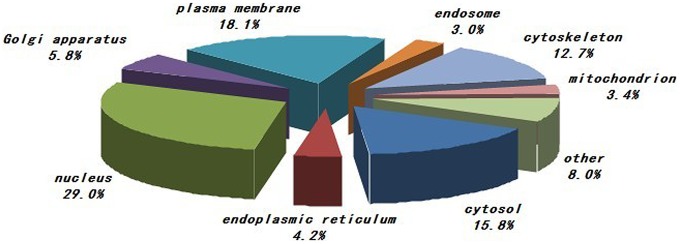
Summary of the cellular components of the PBMCs phosphoproteins characterized by in-gel IEF LC-MS/MS. The most enriched cellular components were nuclear proteins and proteins associated with the plasma membrane, cytosol or cytoskeleton. The information was compiled from Gene Ontology annotations.

**Figure 2:**
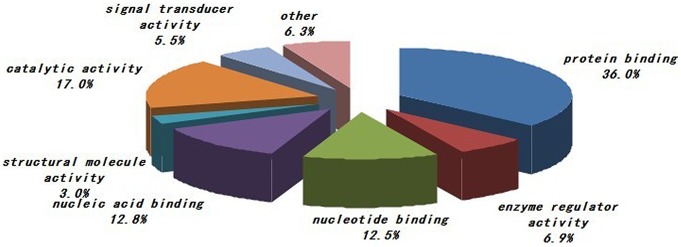
Summary of the molecular functions of the PBMCs phosphoproteins characterized by in-gel IEF LC-MS/MS. The largest group is constituted by protein binding followed by catalytic activity and nucleic acid binding. The information was compiled from Gene Ontology annotations.

**Figure 3:**
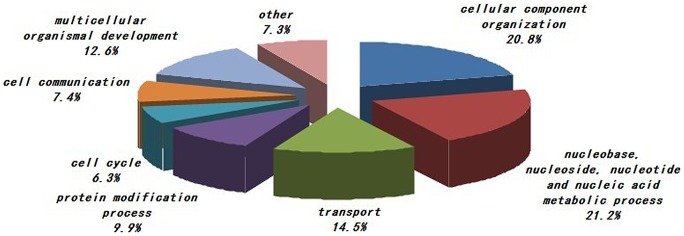
Classification of the characterized PBMCs phosphoproteins based on their involvement in biological processes. The largest group contains proteins related to nucleobase, nucleoside, nucleotide and nucleic acid metabolism. Two other large groups are the proteins involved in cellular component organization and transport. The information was gathered based on Gene Ontology annotations.

The phosphoproteins characterized in the study were evaluated based on their molecular function, biological process and cellular component annotations. As shown in Figure 1, proteins from various cellular components (e.g., the nucleus, plasma membrane, cytosol, cytoskeleton, and Golgi apparatus) were included. The most enriched cellular components were nuclear proteins and proteins associated with the plasma membrane, cytosol or cytoskeleton. Functionally, the phosphoproteins characterized in the study are diverse. We grouped the identified phosphoproteins into several categories based on their molecular functions as annotated in the Swiss-Prot database. The distribution of the phosphoproteins among the various functional categories is shown in Figure 2. The largest group is comprised of proteins with roles in protein binding. The other three largest groups are proteins involved in catalytic activity, nucleic acid binding and nucleotide binding. The distribution of phosphoproteins by biological process is shown in Figure 3. The largest group contains proteins related to nucleobase, nucleoside, nucleotide and nucleic acid metabolism. Two other large groups are the proteins involved in cellular component organization and transport.

### Signaling Pathway Analyses

**Figure 4:**
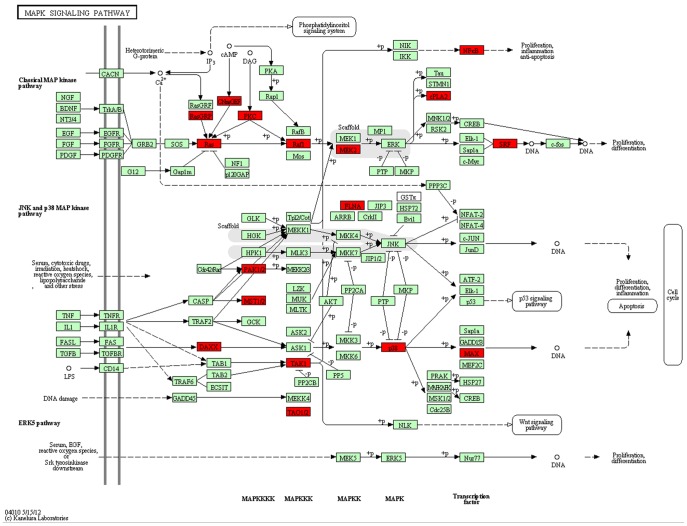
The MAPK signaling pathway showing differentially-expressed gene in SLE patients PBMCs versus healthy controls. Red marks indicate the genes with differential phosphorylation profiles.

We next wanted to determine whether specific pathways are enriched in the set of proteins present in our phosphotyrosine database. Similar to the strategy used for the GO analysis, we mapped differentially modified genes to the KEGG pathway database using GenMAPP v2.1 and then performed a statistical test to identify enriched metabolic pathways, using *P*<0.05 as the standard. We selected 50 metabolic pathways (Table 2). The top KEGG pathway was the MAPK signaling pathway (Figure 4).

**Table 2:** KEEG showing differentially-expressed pathways in SLE patients PBMCs versus healty controls.

Pathway	Gene count	P value
Focal adhesion	16	0.001579
Protein processing in endoplasmic reticulum	11	0.024745
Herpes simplex infection	15	0.001521
Long-term potentiation	7	0.004945
Regulation of actin cytoskeleton	20	5.21E-05
Insulin signaling pathway	10	0.016194
Leukocyte transendothelial migration	9	0.012933
Endocrine and other factor-regulated calcium reabsorption	4	0.038077
Glioma	5	0.037015
Fc gamma R-mediated phagocytosis	15	5.43E-07
Fc epsilon RI signaling pathway	12	9.96E-06
Epithelial cell signaling in *Helicobacter pylori* infection	6	0.014573
*Salmonella* infection	8	0.005302
B cell receptor signaling pathway	7	0.008772
Gap junction	8	0.00664
Spliceosome	14	8.93E-05
ErbB signaling pathway	8	0.005723
Glycolysis/Gluconeogenesis	6	0.011522
Phosphatidylinositol signaling system	9	0.000864
Thyroid cancer	3	0.023349
Pentose phosphate pathway	3	0.020733
Melanogenesis	7	0.038778
Gastric acid secretion	7	0.006928
Starch and sucrose metabolism	5	0.017764
T cell receptor signaling pathway	12	0.000218
Endocytosis	12	0.045153
Pathogenic *Escherichia coli* infection	5	0.017764
Vasopressin-regulated water reabsorption	4	0.025257
Dilated cardiomyopathy	7	0.023862
Natural killer cell mediated cytotoxicity	11	0.00668
Pancreatic secretion	9	0.005007
Ribosome	8	0.007666
MAPK signaling pathway	19	0.00302
Chemokine signaling pathway	17	0.000297
Prostate cancer	8	0.00664
VEGF signaling pathway	7	0.008121
GnRH signaling pathway	12	0.00011
NOD-like receptor signaling pathway	6	0.006232
Long-term depression	8	0.001273
Non-small cell lung cancer	5	0.016332
Shigellosis	6	0.00821
Hypertrophic cardiomyopathy (HCM)	6	0.038708
SNARE interactions in vesicular transport	5	0.002178
RNA transport	13	0.002599
Inositol phosphate metabolism	5	0.024354
Vascular smooth muscle contraction	14	3.07E-05
Adherens junction	6	0.020876
African trypanosomiasis	5	0.002518
Leishmaniasis	6	0.022333
Tight junction	12	0.001512

### Gene Network Analysis

**Figure 5:**
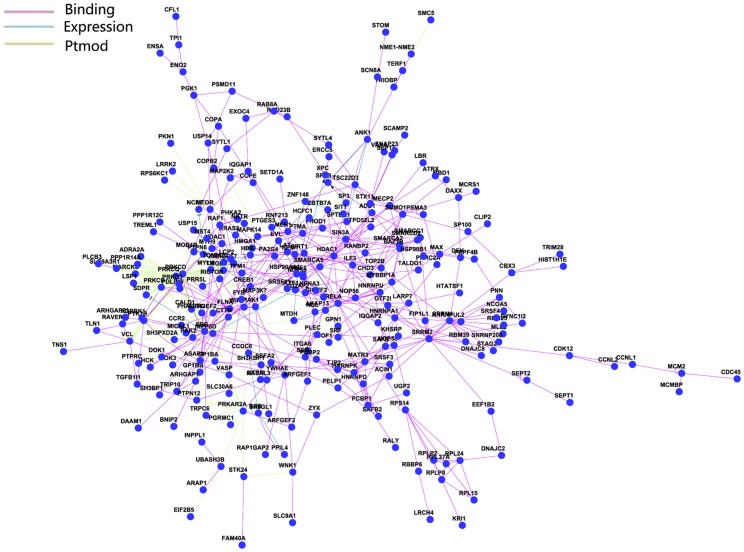
Network analysis of SLE-related genes which were indentified in this analysis. The network can reflect the relationship between genes from the situation as a whole. Blue means expression, gray means binding and purple means ptmod (post-transcription modification).

**Figure 6:**
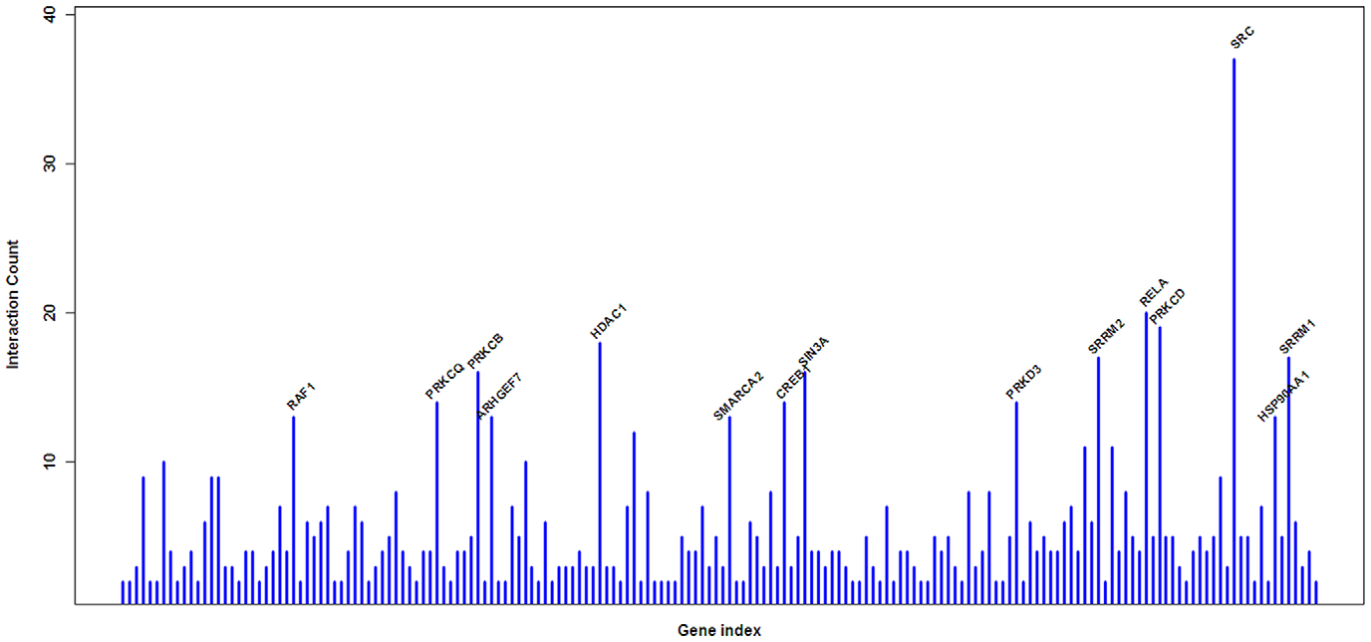
Connectivity analysis of the SLE-related genes. The connectivity of SRC is the highest one in all related-genes.

We integrated the following three different interaction relationships: 1) the gene regulation and protein modification relationships of genes in the KEGG database and other relationships; 2) interaction data from high-flux experiments, such as protein-protein interactions confirmed by yeast two-hybrid; 3) gene-gene interactions described in the literature. Specifically, we downloaded the pathway data from KEGG database and analyzed genome-wide genetic interactions in R (http://www.r-project.org/) and downloaded the KEGGSOAP package (http://www.bioconductor.org/packages/2.4/bioc/html/KEGGSOAP.html). Finally, we integrated the relationships in a gene network (Figure 5). Genes with large numbers of connections were referred to as “hub” genes. Hub genes often play important roles in network stability. We identified SRC, RELA, HDA1C and PRKCD as hub genes in our network (Figure 6).

## Discussion

Protein phosphorylation is the most common posttranslational modification (PTM) in the biosphere [14], [15]. Approximately 30% of proteins can be phosphorylated [16] at threonine, tyrosine and serine residues [17]. Protein phosphorylation becomes disordered when protein kinase or phosphatase activity is overexpressed or inhibited, resulting in abnormal cellular activities and producing cell damage or even cancer [18], [19]. Phosphoproteomics requires powerful analytical technologies and bioinformatics tools. Several recent reviews have summarized the development of various phosphoproteomic methodologies. These methods typically combine different separation strategies with mass spectrometry [20], [21], [22]. The successful application of proteomic technologies to biomedical and clinical research has enabled the discovery of disease-specific biomarkers for diagnosis and treatment monitoring, thus offering insight into the underlying pathologies of diseases and identification of new therapeutic targets.

In this study, we used a phosphorylated peptide reagents kit to enrich the samples for phosphorylated proteins and then combined this technique with mass spectrometry technology. A total of 1035 phosphorylation sites corresponding to 618 annotated genes were identified as differentially modified in SLE compared with normal subjects.

GO analyses showed that the most highly differentially expressed genes were related to nucleic acid metabolism, cellular component organization, transportation, protein modification, cell cycle, cell communication, multicellular organismal development, carbohydrate metabolic process, lipid metabolism and protein translation processes. Nucleic acid metabolism, cellular component organization, transport and multicellular organismal development were the dominant processes. Pathway analysis showed that 50 metabolic pathways are modified in SLE pathogenesis. Notably, MAPK signaling, actin cytoskeleton regulation, chemokine signaling pathway, Fc gamma R-mediated phagocytosis, Herpes simplex infection, spliceosome, vascular smooth muscle contraction and RNA transport process components made up a larger proportion of the genes in these 50 metabolic pathways. The MAPK signaling pathway was highlighted as the most important pathway.

SLE is a chronic autoimmune disorder that is characterized by lymphocyte abnormalities and autoantibody production [23]. Hoffmant showed that immune tolerance defects in the peripheral blood T-lymphocytes of SLE patients related to the abnormal regulation of the MAPK signaling pathway, which directly results in abnormal TCR-mediated intracellular signaling and T lymphocyte function [24], [25]. The MAPK signaling pathway has important functions in many types of mammalian cells. Mitogen-activated protein kinases (MAPKs) are serine and threonine protein kinases that can be activated by phosphorylation in response to extracellular stimuli, such as mitogens, growth factors, cytokines, and osmotic stress [26], [27]. The activation of MAPK pathways has been shown to be a potential pro-inflammatory mechanism in rheumatoid arthritis [28], [29], [30]. During inflammation, MAPK is activated in various immune cells, and its activation is closely related to stress responses and apoptosis [31]. Our results demonstrated that the MAPK signal pathway is abnormally activated in PBMCs from SLE patients, which provided an experimental basis for researching SLE pathogenesis and exploring new therapies. We believe that interventions in or regulation of this signaling pathway may be useful therapies for treating SLE and related diseases.

SRC was the first protein found to have tyrosine protein kinase activity, and its activity is itself regulated by phosphorylation and dephosphorylation. MAPK signaling pathways control multiple physiological processes and are involved in a variety of diseases. Ras, the activating protein upstream of the MAPK pathway, is directly regulated by SRC activity. The activation of the MAPK pathway downstream of Src phosphorylation leads to transcriptional activation. Meanwhile, the inhibition of MAPK pathway activation can partially reverse the effects of SRC protein activity [32]. In particular, as suggested by protein network analysis, genes with many connections within the network were identified as the hub genes. Hub genes often play an important role in the stability of the network. We found that SRC, RELA, HDA1C and PRKCD were the hub genes in our network. These results demonstrated that SRC plays a central role in the stability of the network, suggesting it is important in the pathogenesis of SLE, which provides an experimental basis for researching the pathogenesis of lupus and exploring new treatment methods for SLE.

This experiment thoroughly characterized the phosphorylated protein expression profile in PBMCs of SLE patients. These data will serve as a reference and supplement to help us better understand the pathogenesis of SLE. Furthermore, interventions that modulate the activities of the involved genes and pathways may be able to block or slow the onset and development of SLE.

## Supporting Information

Table S1 **The information of phosphoproteins/phosphopeptides.** The detailed information on the identified phosphoproteins/phosphopeptides according to the mass spectrometric results.
(ZIP)
